# Recurrent Nephrolithiasis in Congenital Cystinuria With a Complete Duplex Collecting System: A Case Report

**DOI:** 10.7759/cureus.112780

**Published:** 2026-07-16

**Authors:** Afrah Mazumder, Tala Al Hanouti, Shooq Faqeeh, Shameema Alam, Laith Suwan, Ismail AlKhalidi

**Affiliations:** 1 Medicine, Health and Medical Services (HMS) Al Garhoud Hospital, Dubai, ARE; 2 Urology, Health and Medical Services (HMS) Al Garhoud Hospital, Dubai, ARE

**Keywords:** congenital anomaly, cystinuria, duplex collecting system, nephrocalcinosis, nephrolithiasis

## Abstract

Cystinuria is a rare autosomal recessive genetic disorder that affects the reabsorption of cystine in the renal tubules. Although cysteine lithiasis is a rare form of nephrolithiasis, it typically presents early in life and is associated with a highly recurrent course and frequent need for repeated surgical interventions. The presence of a duplicated collecting system adds complexity, as it can hinder drainage and make endoscopic access more difficult.

We report the case of a 16-year-old male patient with congenital cystinuria and a complete bilateral duplex collecting system. He presented with recurring colicky pain in his right flank. CT imaging showed lower calyceal cystine stones and bilateral nephrocalcinosis. The initial treatment with extracorporeal shock wave lithotripsy (ESWL) was unsuccessful, leading to staged endoscopic intervention. During ureteroscopy (URS), the tight structure of the ureter and the unfavorable position of the stones made access difficult. This resulted in insufficient fragmentation and the eventual migration of the stone into a deeper calyx that was hard to reach. The procedure was stopped to prevent injury to the ureter. After the surgery, we recommended aggressive hydration and starting an angiotensin-converting enzyme (ACE) inhibitor, with percutaneous nephrolithotomy (PCNL) considered for the future based on how the stones developed.

Cystinuria often requires repeated surgeries, and having a duplex collecting system makes these procedures more challenging and likely to fail. This case illustrates the limitations of URS in complicated anatomical situations. It also emphasizes the need for personalized treatment plans, which may include switching from surgical methods to conservative management when the anatomy makes safe stone removal difficult. Recurrent cystinuria-related kidney stones in a duplex collecting system pose special challenges for diagnosis and treatment. A multidisciplinary approach is crucial to improve outcomes, lower the risks of procedures, and maintain long-term kidney health.

## Introduction

Cystinuria represents an uncommon cause of urinary stone formation [1]. It affects approximately one in 7,000 of the global population [2]. It is an inherited autosomal recessive disorder but can be autosomal dominant with incomplete penetrance, which impairs the reabsorption of cystine and dibasic amino acids (cystine, ornithine, lysine, and arginine (COLA)) in the proximal renal tubules [3], caused by a genetic defect in the b⁰+ amino acid transporter, with two known mutations involving SLC3A1 and SLC7A9, although up to 5% of patients do not have an identified mutation [4]. Cystine's low solubility results in its accumulation in urine, ultimately causing the formation of calculi [5]. Cystine stones make up approximately 1%-2% of all kidney stones, yet they account for about 6%-8% of urinary stones occurring in the pediatric age group [3]. The initial medical management includes increasing daily fluid intake, along with sodium- and protein-limiting dietary changes. Urine alkalinizers such as potassium citrate and cystine-binding thiol agents are taken into consideration if these methods prove ineffective [6]. Despite receiving medical treatment, individuals with cystine stones typically necessitate between 3.1 and 4.2 surgical interventions during a follow-up duration of five to eight years [1].

Small stones may be treated with extracorporeal shock wave lithotripsy (ESWL), although cystine's resistance should be considered due to its high hardness and strong crystal cohesion, which limit effective fragmentation and result in larger residual fragments and lower stone-free rates compared with other stone compositions [3,7]. While 2 cm is generally considered the upper size limit for ESWL in most stone types, consensus guidelines suggest a lower threshold for cystine stones, with 10 mm being the upper limit for ESWL in lower pole cystine calculi, and for stones measuring 10-20 mm in other renal locations, ESWL is considered a third-line option after ureteroscopy (URS) and percutaneous nephrolithotomy (PCNL) [3]. Cystinuria commonly presents with early-onset nephrolithiasis and is associated with a high recurrence rate of up to 60%, as reported in long-term studies involving mixed adult and pediatric cohorts with extended follow-up, and due to its impact on the quality of life and the risk of chronic kidney disease, cystinuria requires lifelong management [8].

Nephrocalcinosis is the accumulation of calcium salts in the renal parenchyma (often medullary), as opposed to nephrolithiasis and urolithiasis, which are the presence of stones in the collecting system. A small proportion of genetic disorders linked to nephrolithiasis and nephrocalcinosis are due to metabolic abnormalities, including cystinuria [9]. Although nephrocalcinosis is less common in cystinuria, its presence can complicate clinical management by shifting care from a primarily stone-removal, procedural approach to a long-term medical and metabolic strategy focused on underlying tubular disorders and the prevention of chronic kidney disease progression.

Embryologically, the duplication of the urinary system arises when the ureteric bud divides abnormally. Partial division results in a partially duplicated kidney accompanied by a bifid ureter, whereas complete division leads to the formation of a fully duplicated (double) kidney, which may drain through either a bifid ureter or two entirely separate ureters. These anatomical variations can alter drainage patterns and access routes, thereby increasing procedural complexity during endourological and surgical interventions [10,11].

We report a case of recurrent cystine stones in a patient with congenital cystinuria and a complete bilateral duplex collecting system, emphasizing the complexity of treatment.

## Case presentation

A 16-year-old male patient with a previously diagnosed history of congenital cystinuria and recurrent cystine nephrolithiasis presented with right renal colic. He also reported having a congenital bilateral complete duplicated collecting system. There was no significant family history of urolithiasis or metabolic disorders.

The patient had been managed conservatively with urinary alkalinization; however, this approach had been associated with recurrent stone-related procedures in the past. Since July 2020, he had experienced multiple episodes of intermittent dull, colicky right flank pain. The current report focuses on his most recent episode. In July 2025, he presented with intermittent colicky right flank pain without gross hematuria, fever, or constitutional symptoms. At the presentation, he was receiving only urinary alkalinization therapy.

**Figure 1:**
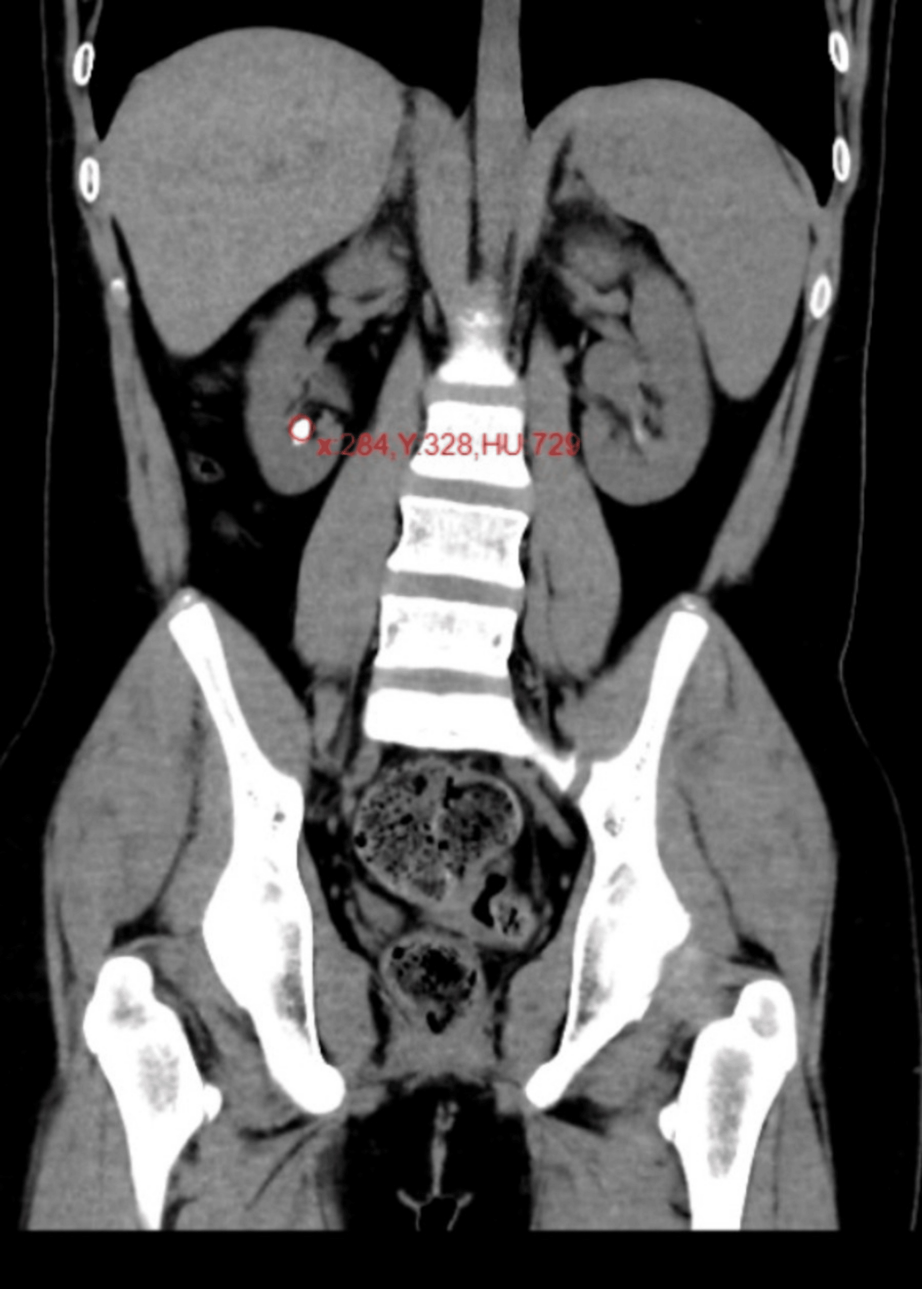
CT scan showing a right lower calyceal stone measuring 10 × 6 mm, with a CT attenuation of approximately 742 Hounsfield units (HU). Although non-contrast CT is the standard imaging modality for urolithiasis, contrast-enhanced CT was performed in this patient to better delineate the complex renal collecting system anatomy, given the known duplex ureter and the extensive history of prior urological interventions [2]. Contrast imaging aided in procedural planning and the assessment of the collecting system prior to management.

A contrast-enhanced CT scan of the abdomen revealed a 10 × 6 mm calculus within the right lower calyx with a CT attenuation of approximately 742 Hounsfield units (HU). An additional smaller calculus measuring 2.7 mm was noted in the right renal lower pole with a CT density of 283 HU. Multiple bilateral punctate calcifications at the corticomedullary junction were also observed, suggestive of nephrocalcinosis (Figure 1).

Managed by right ESWL, after two months, cystourethroscopy visualized with right retrograde pyelography and right URS with right double-J (DJ) stent insertion was done in the lower system, followed by cystourethroscopy and the right DJ stent removal and right retrograde pyelography with flexible URS with laser lithotripsy.

Cystoscopic evaluation demonstrated two separate ureteric orifices on each side (four ureteric orifices in total), confirming a bilateral complete duplicated collecting system. Ureteroscopic access was obtained through the right lower-moiety ureter, and a DJ stent was placed in the same ureter at the conclusion of the procedure.

During the initial URS procedure, the right ureter was found to be tight, rendering it unsafe to proceed with flexible ureterorenoscopy due to the significant risk of ureteric injury. Consequently, a DJ stent was inserted in the right lower moiety. The subsequent attempt at flexible ureterorenoscopy and laser lithotripsy was unsuccessful, as the complete fragmentation of the stone could not be achieved due to these anatomical difficulties (Figure 1).

**Figure 2:**
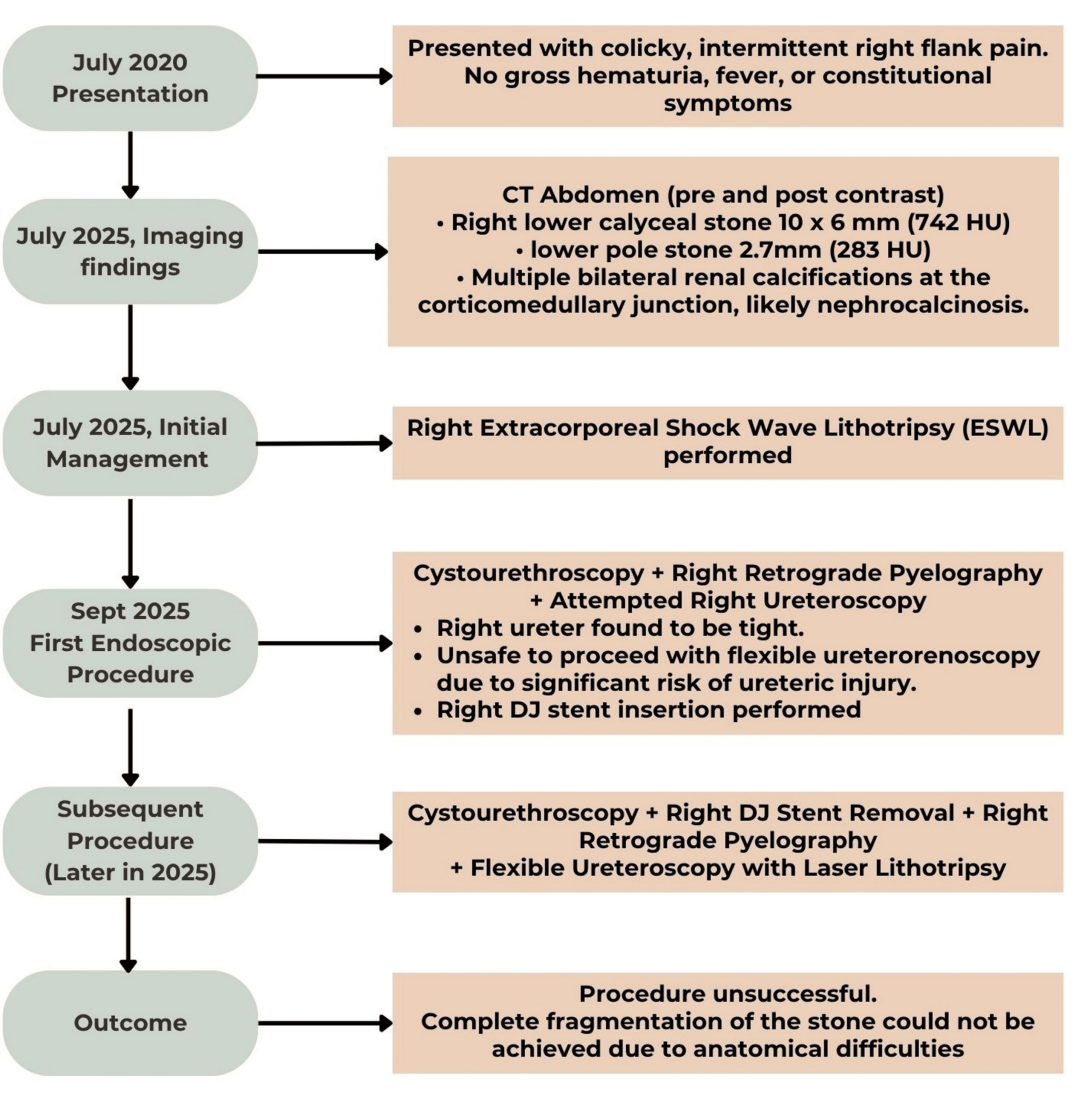
Timeline of the case presentation along with interventions performed. HU: Hounsfield unit; DJ: double J This figure has been created by the authors using Microsoft PowerPoint (Microsoft Corp., Redmond, WA, USA).

Metabolic workup was done to assess the biochemistry profile, which revealed no pathological findings. The slightly low serum creatinine depicted was clinically insignificant given the concurrent, highly favourable estimated glomerular filtration rate (eGFR) of 143 mL/min/1.73 m2. The overall metabolic, electrolyte, and renal profile was within normal physiological parameters (Table 1).

**Table 1:** Laboratory serum workup

Test	Result	Reference Range
Calcium	9.9 mg/dL	8.4-10.2
Creatinine	0.63 mg/dL (low)	0.70-1.10
Glucose, random	91 mg/dL	75-140
Albumin	4.3 g/dL	3.20-4.50
Phosphorous	4.7 mg/dL	2.7-4.9
Urea, serum	36 mg/dL	18-45
Bicarbonate	24 mmol/L	22-29
Sodium	139 mmol/L	136-145
Potassium	4.2 mmol/L	3.5-5.1
Chloride	100 mmol/L	97-107
Estimated glomerular filtration rate	143 mL/minute/1.73 m^2^	-

Further workup revealed markedly elevated 24-hour total cystine levels compared with the laboratory reference range (Table 2).

**Table 2:** Twenty-four-hour urine panel with laboratory reference values. The 24-hour urine collection was obtained postoperatively and before the initiation of captopril therapy ᵃTarget values established based on clinical guidelines outlined by Eisner et al. [12]; ᵇLaboratory reference value; ^c^Reference diagnostic target as cited in the studies by Eisner et al. [12] and Azer et al. [13].

Laboratory Parameter	Patient Value	Reference Value
Urine volume	3.07 L/24 hours	Target ≥2.5-3.0 L/24 hours for adults; ≥2.0 L/24 hours for childrenᵃ
24-hour urine cystine	174.05 mg/24 hours	Less than 38.10 mg/24 hoursᵇ
Calculated urinary cystine concentration	56.7 mg/L	Therapeutic target: <250 mg/L^c^

Radiological description of the duplex system

**Figure 3:**
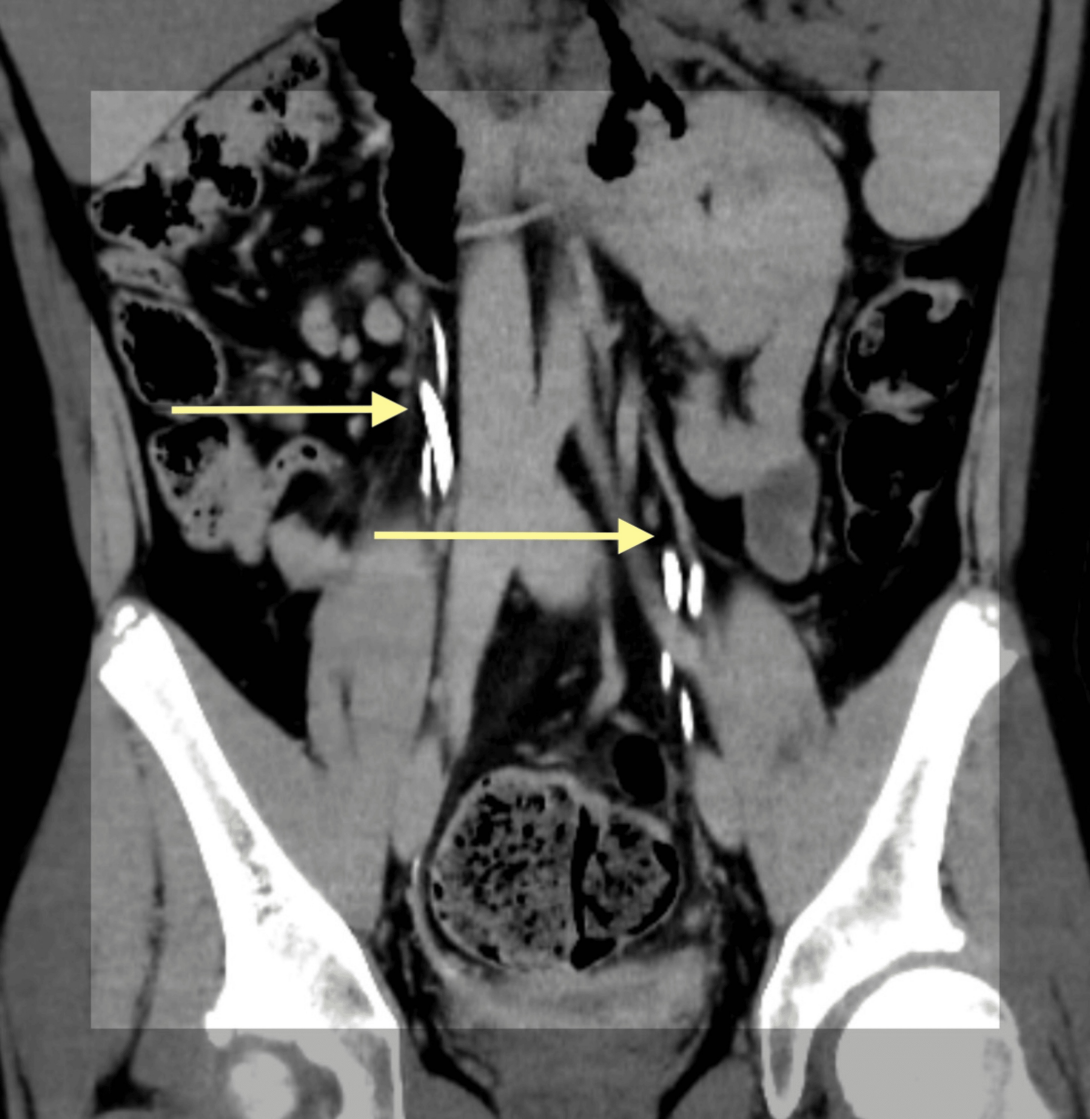
CT scan revealed a bilateral complete duplex collecting system (yellow arrow).

The bilateral complete duplication of the renal collecting systems is present, with two ureters on each side draining separately into the urinary bladder via two distinct ureteric orifices bilaterally (four ureteric orifices in total), without evidence of distal ureteral confluence (Figure 3).

Challenges encountered and outcomes

The patient had a previous history of multiple procedures (four previous percutaneous nephrolithotomies, three sessions of ESWL, one DJ ureteral stent placement, and one prior URS).

The previously placed DJ stent was removed. A ureteroscope was then advanced retrogradely through the ureter into the renal pelvis. Upon inspection, a calculus was located within the lower calyx of the kidney. Initial visualization of the stone was challenging due to its position within the calyx. After several attempts, adequate visualization was achieved, and the laser fiber was aligned with the stone. Nevertheless, when we started ablating the stone, this caused the stone to migrate deeper into the lower calyx, rendering it inaccessible thereafter. 

In succession, multiple maneuvers were attempted in a controlled manner, systematically changing the patient’s position to facilitate stone repositioning while ensuring patient safety. Gentle flank percussion was also employed to mobilize the embedded fragment and improve visualization. Despite these efforts, the stone remained large and deeply impacted; consequently, the decision was made to terminate the procedure and leave the stone in situ.

A postoperative plan was made for the patient, which was as follows: high-volume hydration (3 liters of water daily) and referral to the nephrology department for suggested captopril therapy for its sulfhydryl (thiol) cystine-binding properties [11]; follow-up appointments to monitor the size of the calculi; and, within a six-month interval, a repeat PCNL would be considered with due clinical judgment.

## Discussion

Cystinuria is the most common hereditary cause of nephrolithiasis in children, accounting for approximately 6%-8% of pediatric urolithiasis cases. Patients with cystine stones have been found to have less favorable health-related quality of life than those without cystine stones, and there is considerable diversity in how cystinuria presents clinically. Cystinuria patients are more likely to require recurring surgical treatments for stones, which raises their risk of chronic kidney disease, renal insufficiency, and end-stage renal disease. The goal of medical intervention is to render cystine soluble in urine [12]. To the best of our knowledge, this is the first reported pediatric case of cystinuria associated with a bilateral complete duplicated collecting system.

The primary goal of medical management is to increase urinary cystine solubility, primarily through high fluid intake and urinary alkalinization. In adults, this requires a robust fluid intake to achieve a minimum desired urine output of 2.5 to 3.0 L per day, while the target urine production in children is 2.0 L per day. Dietary modifications, including reduced sodium intake and moderation of methionine-rich foods, may help reduce cystine excretion, although intake should not fall below physiological requirements [12].

To assess the efficacy of these conservative measures, serial 24-hour urine examinations are crucial. However, clinically, a distinct separation must be maintained between the laboratory reference values used for diagnosis and the concentration-based treatment targets used in management [13].

Total daily cystine excretion (measured in mg/24 hours) serves primarily as a diagnostic marker; physiological excretion in healthy subjects is typically less than 30 mg/24 hours, while cystinuria patients often excrete more than 300 mg/24 hours [14]. Conversely, therapeutic success depends entirely on urinary cystine concentration (measured in mg/L), as cystine precipitates out of solution when its concentration exceeds its solubility limit [13].

The clinical management target is to maintain a urinary cystine concentration of less than 250 mg/L at an optimal urinary pH of greater than 7.0 [12,13]. If conservative fluid and dietary approaches fail to achieve this concentration target, alkali treatment with potassium citrate, administered two or three times per day, is recommended to raise and sustain a 24-hour urine pH between 7.0 and 7.5 [11].

To properly contextualize these metabolic metrics, the timing of the patient's 24-hour urine collection relative to clinical milestones is noted. Our sample collection was performed after the initiation of urinary alkalinization therapy and after the surgical intervention. Furthermore, the sample was obtained prior to the initiation of captopril therapy.

In this patient's case, while the total 24-hour cystine excretion remains elevated beyond the normal diagnostic reference range at 174.05 mg/24 h, their exceptional urine volume of 3.07 L/24 h successfully dilutes the solute. This yields a calculated urinary cystine concentration of 56.7 mg/L, well below the 250 mg/L therapeutic threshold required to prevent crystallization.

Standard management of cystinuria-associated nephrolithiasis includes high fluid intake, urinary alkalinization, and thiol-binding agents such as tiopronin or D-penicillamine. Captopril contains a sulfhydryl group capable of forming soluble mixed disulfide complexes with cystine. The recommended doses for children are 1.5-6 mg/kg/day, max dose 150 mg/day; and for adults is 75-150 mg/day with a frequency of 3 times per day [14]. It is not considered standard therapy for cystinuria; its potential benefit is attributed to its thiol-binding properties rather than its angiotensin-converting enzyme inhibitory effect. Sulfhydryl-containing compounds, including captopril, can convert cystine to more soluble disulfides [11].

When it comes to surgical management, ESWL may be explored for pediatric patients given the disease's highly recurring nature [5], as in our patient.

Duplicated collecting systems are the most common congenital upper urinary tract anomaly and are often incidentally detected. While many cases remain asymptomatic, they may be associated with recurrent urinary tract infections, vesicoureteral reflux, obstruction, ureterocele, and urolithiasis. The reported incidence of stone formation in duplex systems ranges from 4% to 20% [15].

Our patient presented with right lower calyceal stones as well as a smaller stone in the lower pole. His presentation was further complicated by multiple renal calcifications consistent with nephrocalcinosis. It is critical to evaluate the hazards of both intervention and non-intervention in pediatric patients, including the possibility of chronic renal failure, hypertension, and loss of kidney function. Endoscopic procedures such as retrograde intrarenal surgery or PCNL should aim to remove all stone fragments because of the high recurrence rate in children with cystinuria, as even millimetric residual stones may regrow and aggregate [5]. PCNL was recommended as a treatment option, but the patient's parents rejected it because he had already had four PCNL surgeries. Therefore, ESWL was selected as the initial treatment after the team and parents examined the various treatment alternatives.

When the initial treatment didn’t work, we hoped that the follow-up surgery to clear the blockage and restore proper drainage would be more successful. Unfortunately, despite our best efforts, the stone moved deeper into the lower calyx, forcing us to stop the surgery early. 

Captopril was suggested as a potential adjunctive therapy as a sulfhydryl-containing agent and due to its availability at our institution. The patient was subsequently referred to the nephrology department for ongoing metabolic management; however, he was lost to follow-up after returning to his home country. Therefore, the initiation, adherence, and clinical effect of any further pharmacologic therapy remain unknown. Prior to loss to follow-up, no recurrent stone-related symptoms, urinary tract infections, or procedure-related complications were reported. 

The diagnosis of cystinuria had been established in the patient’s home country prior to presentation; however, original medical records were not available for review. Stone composition analysis, genetic testing for SLC3A1 and SLC7A9, and comprehensive metabolic evaluation were not performed at our institution. In addition, family screening data were not available. These limitations preclude full characterization of the metabolic profile and contribute to uncertainty regarding the extent of associated abnormalities such as nephrocalcinosis. Furthermore, long-term imaging and metabolic follow-up were not available due to loss to follow-up.

These limitations highlight the challenges of managing complex stone disease in patients with rare congenital urinary tract anomalies, particularly when complete metabolic and longitudinal data are unavailable.

## Conclusions

This case highlights the complexity of managing recurrent cystine nephrolithiasis in a pediatric patient with a bilateral complete duplicated collecting system. Anatomical variation, recurrent stone disease, and the need for staged intervention contributed to technical challenges during endourological management.

Cystinuria requires individualized management combining medical and surgical strategies, with careful consideration of anatomical and metabolic factors that may influence outcomes. This single case demonstrates technical difficulty in one patient, but cannot definitely state ureteroscopic failure, effectiveness of captopril, or that PCNL is a future option. This case underscores the importance of comprehensive pre and postoperative evaluation and multidisciplinary planning in complex pediatric stone disease. Further studies are needed to better define optimal management strategies in patients with cystinuria and associated congenital urinary tract anomalies.
